# Hepatic encephalopathy associated with extensive hepatic diaphragmatic herniation in an adult horse

**DOI:** 10.1186/s12917-026-05752-w

**Published:** 2026-07-31

**Authors:** Markyson Tavares Linhares, Isabelle Vieira de Sousa, Lilian Rayanne de Castro Eloy, José Machado de Oliveira Neto, Luis Jhordy Alfaro Quillas, Daniel Guimarães Ubiali, Lucas Rannier Ribeiro Antonino Carvalho, Ricardo Barbosa Lucena

**Affiliations:** 1 PatoBiology Laboratory, Universidade Federal da Paraíba, Areia, Paraíba 58397-000 Brazil; 2 Section of Anatomic Pathology, Institute of Veterinary Medicine, Universidade Federal Rural do Rio de Janeiro, Rio de Janeiro, Seropédica Brazil; 3 Department of Physiology and Pharmacology, Karolinska Institutet, 5B, Solnavägen 9, Stockholm, S-171 77 Sweden

**Keywords:** Cholestasis, Encephalopathy, Equine, Fibrosis, Liver, Necropsy

## Abstract

**Background:**

Diaphragmatic hernia in horses is a rare condition, most commonly presenting with signs of colic and, less frequently, respiratory compromise. Hepatic herniation through a diaphragmatic defect with secondary hepatic failure and encephalopathy has rarely been documented in horses.

**Case presentation:**

A 5-year-old Mangalarga Marchador stallion was evaluated for progressive neurological deterioration, including obtundation, head pressing, ataxia, and circling. Serum biochemistry revealed severe hepatobiliary dysfunction, characterised by increased gamma-glutamyl transferase and alkaline phosphatase activities, hyperbilirubinaemia and hypertriglyceridaemia. The horse died within 12 h of admission. Post-mortem examination revealed a chronic pleuroperitoneal diaphragmatic defect with herniation of the left lateral, left medial, and quadrate hepatic lobes into the thoracic cavity. The herniated portion showed severe atrophy, haemorrhagic necrosis, cholestasis, and ductular proliferation, while the non-herniated hepatic mass exhibited bridging fibrosis and nodular regenerative remodelling. Histopathological examination of the brain confirmed the presence of Alzheimer type II astrocytes in the cerebral cortical grey matter, consistent with hepatic encephalopathy.

**Conclusions:**

This case documents an unusual manifestation of equine diaphragmatic hernia in which extensive chronic hepatic incarceration resulted in severe hepatobiliary dysfunction and hepatic encephalopathy. The findings suggest that diaphragmatic hernia with hepatic involvement should be considered in the differential diagnosis of horses presenting with severe hepatopathy and neurological signs compatible with hepatic encephalopathy, particularly when classical signs of colic are absent.

## Background

Diaphragmatic hernias are defined as the displacement of abdominal organs or tissues into the thoracic cavity through a defect in the diaphragm [1]. In horses, this condition is considered rare, accounting for approximately 1–8% of exploratory laparotomies [2, 3]. Diaphragmatic defects may be congenital or acquired and are anatomically classified as pleuroperitoneal or pericardioperitoneal, depending on their location relative to the pericardium [2, 4].

The most common clinical presentation of diaphragmatic hernia in horses involves herniation of gastrointestinal viscera, typically resulting in signs of colic and, in some cases, respiratory compromise [5, 6]. Hepatic herniation through a diaphragmatic defect is substantially less common and has been described only rarely in the equine literature [7]. Because clinical signs may be variable and non-specific, diagnosis of diaphragmatic hernia ante mortem remains challenging, and cases involving hepatic tissue may go unrecognised when classical colic signs are absent [8].

Hepatic encephalopathy is a well-recognised neurological syndrome secondary to severe hepatic dysfunction in horses, typically developing when more than 70–80% of functional hepatic reserve is lost [9]. Clinically, affected horses may show depression or obtundation, behavioural changes, head pressing, ataxia, circling, apparent blindness, seizures, recumbency and coma [9]. Clinicopathological abnormalities usually reflect severe hepatobiliary dysfunction and may include hyperbilirubinaemia, increased serum activities of hepatocellular and cholestatic enzymes such as aspartate aminotransferase (AST), gamma-glutamyl transferase (GGT) and alkaline phosphatase (ALP), altered bile acid concentrations, reduced blood urea nitrogen, hypoalbuminaemia, metabolic disturbances, and hyperammonaemia [9–11]. However, no single laboratory abnormality is diagnostic, and interpretation requires integration with clinical signs and evidence of severe hepatic disease [9, 11]. In horses, hepatic encephalopathy may result from different causes of severe hepatic dysfunction or reduced hepatic functional reserve, including toxic hepatopathies, portosystemic vascular disorders, infectious or inflammatory hepatopathies and chronic fibrosing liver diseases [10–12].

This report describes the clinical, laboratory, gross, and histopathological findings of an adult horse with extensive chronic hepatic herniation through a chronic pleuroperitoneal diaphragmatic defect, associated with severe hepatobiliary dysfunction and hepatic encephalopathy.

## Case report

A 5-year-old Mangalarga Marchador stallion was referred for *post-mortem* examination following death after a short clinical course characterised by progressive neurological deterioration. Clinical information was obtained retrospectively from the referring veterinarian and available medical records.

According to the clinical history, the horse had no prior reported episodes of colic, weight loss, or other clinical signs before transport. The animal had been transported by road over approximately 1,900 km from southeastern to northeastern Brazil and was kept in isolation following arrival. Approximately one week after transport, progressive behavioural and neurological abnormalities were observed, initially characterised by restlessness and excitability, followed by locomotor difficulty and ataxia. The horse had not been examined by a veterinarian during transport or immediately after arrival; therefore, the precise onset of clinical signs during this period could not be established.

On admission to the Equine Veterinary Hospital in Gravatá, Pernambuco, Brazil, the horse weighed 400 kg and had a body condition score of 5/9. Rectal temperature was 37.6 °C. Tachycardia and tachypnoea were noted on physical examination; however, exact values were not available in the medical records provided. Capillary refill time was not recorded. Oral mucous membranes were mildly icteric. Mild dyspnoea was evident, characterised by increased respiratory rate and slight nostril flare, without abdominal effort.

Complete blood count revealed no clinically relevant abnormalities. Serum biochemistry demonstrated a pattern consistent with severe hepatobiliary dysfunction (Table 1). Serum bile acids and glutamate dehydrogenase (GLDH) were not measured, as these were not available at the referring institution.

**Table 1:** Serum biochemical findings in the horse with hepatic diaphragmatic herniation

Parameter	Result	Reference interval	Unit
GGT	296	4–44	U/L
ALP	554	143–395	U/L
AST	650	116–487	U/L
Total bilirubin	111.2	3.4–39.3	µmol/L
Triglycerides	3.64	0.11–0.56	mmol/L
Total cholesterol	4.06	1.29–3.9	mmol/L
Glucose	185.4	62–134	mg/dL
Lipase	39	1–20	U/L

*GGT* gamma-glutamyl transferase, *ALP* alkaline phosphatase, *AST* aspartate aminotransferase

Neurological examination revealed marked obtundation, intermittent disorientation, and decreased responsiveness to external stimuli, accompanied by mild generalised muscle tremors. The horse exhibited head pressing, ataxia, motor incoordination, and a wide-based stance in all four limbs. Transient cortical blindness was suspected based on repeated collision with objects and an absent menace response, despite preservation of the pupillary light reflex. No signs of abdominal pain were reported at any point during the clinical course.

During hospitalisation, the neurological condition deteriorated rapidly. The horse developed tongue protrusion, ineffective prehension and chewing movements, bruxism, and circling to the right. Intermittent generalised seizures ensued, followed by progressive stupor, prolonged recumbency, coma, and death within 12 h of admission. Supportive treatment was initiated; however, specific details were not available from the medical records.

A complete *post-mortem* examination was performed. Tissue samples from internal organs, brain, spinal cord, and bone marrow were fixed in 10% neutral buffered formalin, routinely processed, embedded in paraffin, sectioned at 4 μm, and stained with haematoxylin and eosin (H&E). Additional liver sections from both hepatic portions were stained with Masson’s trichrome to assess fibrosis and Perls’ Prussian blue to evaluate iron deposition. An age-matched equine liver without histological evidence of hepatic disease was used as a negative control for Perls’ staining.

**Fig. 1:**
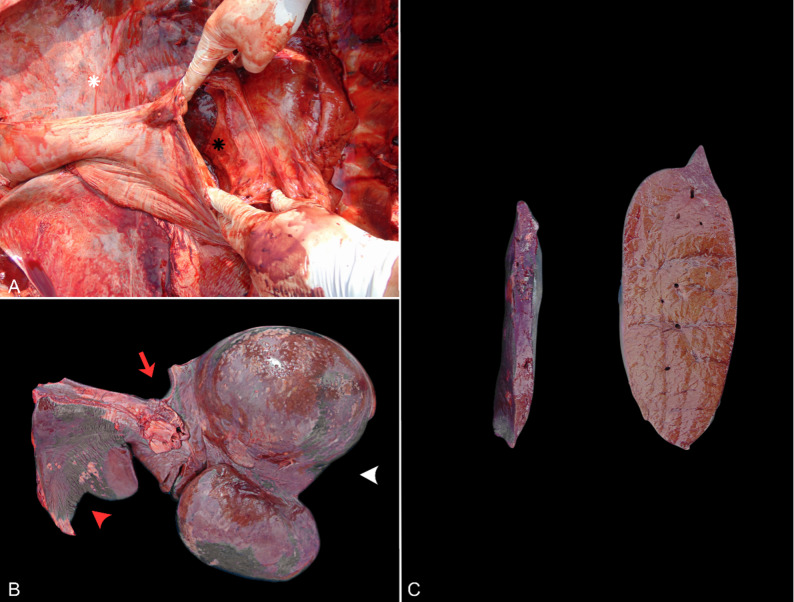
Extensive hepatic herniation through a diaphragmatic defect in a horse. **A** Diaphragm (white asteristic) presenting large defect (black asteristic) with smooth, rounded fibrous margins involving the left dorsal tendinous portion of the diaphragm, creating a pleuroperitoneal communication. **B** Liver removed at necropsy showing marked asymmetric large nodules between the herniated and non-herniated hepatic portions. The herniated portion, comprising the left lateral, left medial, and quadrate lobes, is severely atrophic, flattened, and firm (red arrowhead). The non-herniated right hepatic lobe, which remained within the abdominal cavity, is markedly enlarged and globoid (white arrowhead). A constricted transitional area at the hernial ring is also evident (red arrow). **C** Cut surfaces of the herniated and non-herniated hepatic portions. The herniated portion (left) is markedly reduced in volume, firm, and dark red-brown. The non-herniated right hepatic lobe (right) is enlarged, firm, and yellow-brown with an irregular nodular appearance

Externally, multiple subcutaneous haematomas were observed. Approximately 3 L of serosanguineous fluid was present in the thoracic cavity; laboratory analysis of this fluid was not performed. The diaphragm exhibited a 12 cm defect with smooth, rounded, white fibrous margins involving the left dorsal tendinous portion, establishing a pleuroperitoneal communication (Fig. 1A). No hernial sac was identified.

Through this defect, the left lateral, left medial, and quadrate hepatic lobes were displaced into the thoracic cavity, comprising the majority of the total hepatic mass. The liver was markedly asymmetric: the herniated portion was severely atrophic, flattened, and firm, while the non-herniated right hepatic lobe remaining in the abdominal cavity was markedly enlarged, globoid, and irregular, with multifocal to coalescing pale areas on the capsular surface (Fig. 1B). A constricted transitional area was evident adjacent to the hernial ring, consistent with chronic compression at the site of herniation. On cut surface, both hepatic portions were firm; the herniated portion was dark red-brown and markedly reduced in volume, whereas the non-herniated portion was yellow-brown with a nodular appearance (Fig. 1C). Approximately 200 mL of serosanguineous fluid was present in the pericardial sac; the lungs were diffusely congested, oedematous, and glistening, with pleural adhesions between the caudal pulmonary lobes and the diaphragm adjacent to the hernial defect. Abundant foamy fluid was present in the tracheal lumen, consistent with pulmonary oedema.

**Fig. 2:**
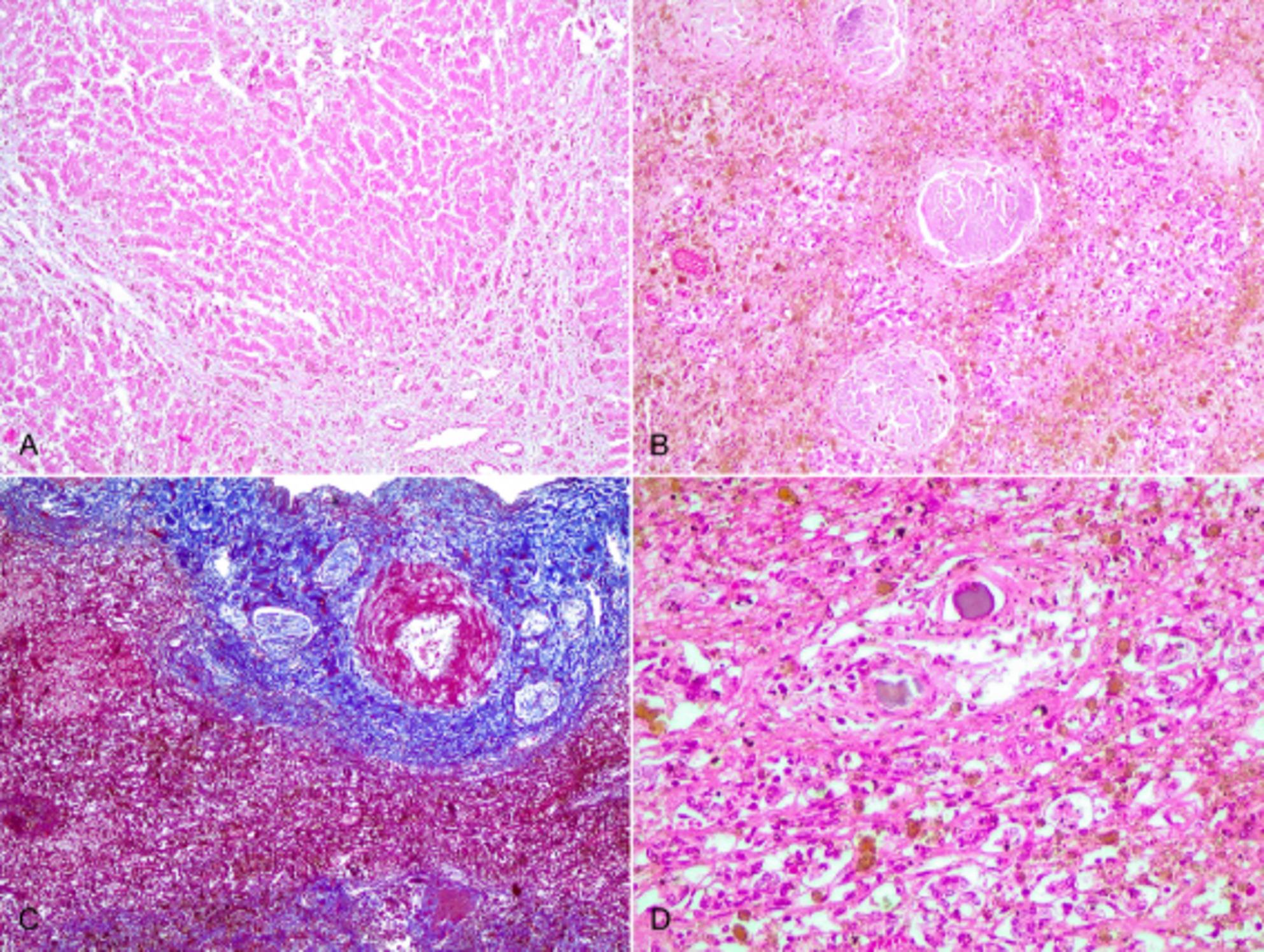
Hepatic histopathological changes associated with extensive diaphragmatic herniation. **A** Non-herniated hepatic portion showing bridging fibrosis with nodular regenerative remodelling and hepatocellular cytoplasmic vacuolation. Haematoxylin and eosin (H&E), 10×. **B** Herniated hepatic portion showing marked cholestasis, ductular proliferation, brown-orange bile pigment and vascular wall hypertrophy. H&E, 20×. **C** Herniated hepatic portion with marked parenchymal atrophy and collapse associated with extensive fibrosis, highlighted in blue by Masson’s trichrome stain. 10×. **D** Herniated hepatic portion showing severe cholestasis, ductular reaction and intraductal basophilic bile concretions compatible with microliths/choleliths. H&E, 20×

**Fig. 3:**
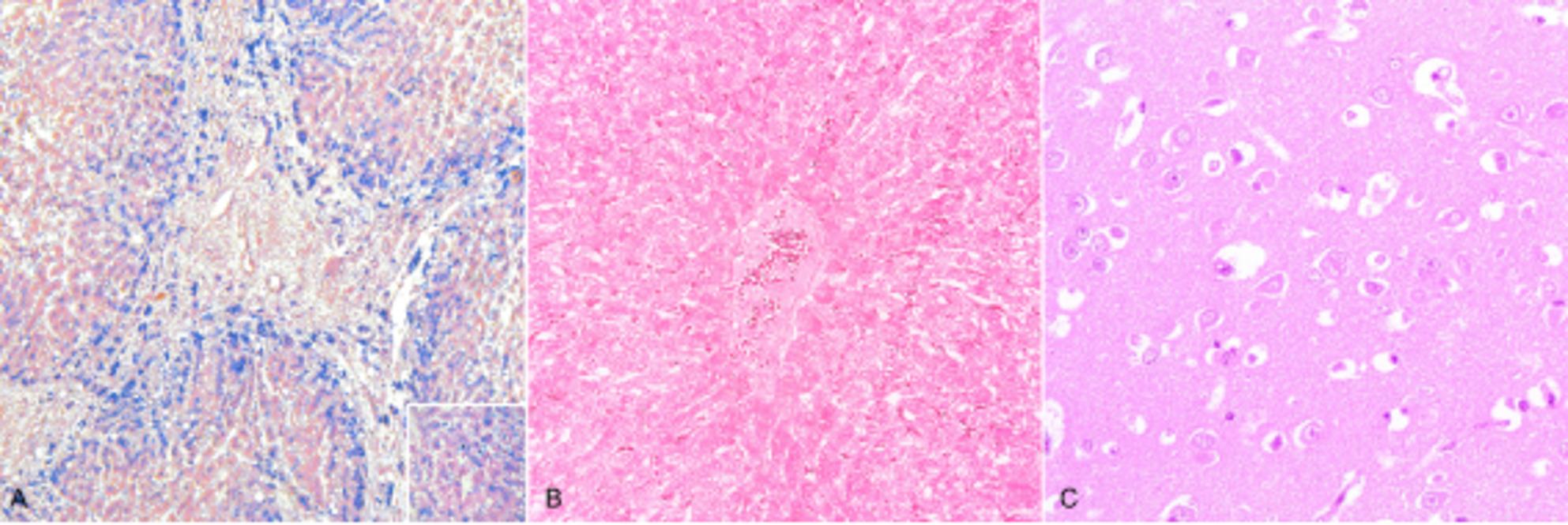
Hepatic haemosiderin deposition and cerebral changes associated with hepatic encephalopathy in the affected horse. **A** Abundant iron-positive deposits within the hepatic parenchyma (Perls’ Prussian blue 10×). **B** Age-matched control equine liver without comparable iron-positive deposition (Perls’ Prussian blue 10×). **C** Swollen astrocytes with enlarged nuclei and margined chromatin, arranged in pairs or small clusters of up to four cells (Alzheimer type II cells) on the cerebral cortex. (Haematoxylin and Eosin, 40×)

Histologically, the non-herniated hepatic portion exhibited diffuse bridging fibrosis with formation of regenerative nodules, consistent with advanced cirrhotic remodelling. Hepatocytes within these nodules showed cytoplasmic vacuolation with peripheral nuclear displacement, consistent with lipid-type vacuolar degeneration (Fig. 2A). The herniated hepatic portion showed marked cholestasis, characterised by orange-brown bile pigment within canaliculi and sinusoidal spaces, together with biliary ductular proliferation in fibrotic portal areas (Fig. 2B). Multifocal haemorrhages, hepatocellular necrosis, and severe parenchymal atrophy with replacement by fibrous connective tissue were also present, highlighted by Masson’s trichrome staining (Fig. 2C). Some proliferating bile ducts contained basophilic intraluminal concretions compatible with bile microliths (Fig. 2D). In areas of atrophy, fibrosis, and haemorrhage, numerous macrophages and Kupffer cells contained coarse golden-brown pigment that stained strongly with Perls’ Prussian blue, confirming haemosiderin deposition (Fig. 3A), a finding not present in the age-matched control liver (Fig. 3B). Hepatic venous profiles showed marked smooth muscle hypertrophy, consistent with chronic vascular remodelling.

In the cerebral cortical grey matter, astrocytes with large pale nuclei and prominent nucleoli, consistent with Alzheimer type II astrocytes, were identified (Fig. 3C). No inflammatory, necrotising, malacic, or infectious-type lesions were identified in the central nervous system. Pulmonary sections showed alveolar spaces filled with pale eosinophilic proteinaceous fluid, consistent with oedema. No relevant histopathological lesions were observed in other examined organs.

## Discussion

The present case represents an unusual manifestation of diaphragmatic hernia in an adult horse, characterised by extensive hepatic herniation associated with severe hepatobiliary dysfunction and hepatic encephalopathy. Although diaphragmatic hernias in horses classically present with colic and, in some cases, respiratory compromise, the clinical course in this horse was dominated by progressive neurological abnormalities [13, 14].

This difficulty in clinical recognition was compounded by the chronic nature of the diaphragmatic defect. Diaphragmatic hernias in horses may be congenital or acquired. Congenital defects are usually interpreted as developmental abnormalities of the diaphragm, whereas acquired defects may result from blunt trauma, increased intra-abdominal pressure, or traumatic events occurring during birth or early life, including rib fractures associated with dystocia or neonatal trauma [4, 15]. At necropsy, the defect in this horse was located in the left dorsal tendinous portion of the diaphragm and exhibited smooth, rounded, fibrous margins, features more consistent with a long-standing lesion than with an acute traumatic rupture. Although no hernial sac was identified, this does not exclude a congenital origin, as many equine diaphragmatic defects lack a true sac and may be classified as false hernias [4]. Congenital diaphragmatic defects in horses have been reported most frequently in the left dorsal tendinous portion, presenting as chronic, smooth-edged openings [15]. Therefore, the anatomical location and gross morphology of the defect in this horse supported a presumptive developmental or early-life origin. The age of five years at clinical presentation does not exclude this possibility, because congenital or perinatal diaphragmatic defects may remain clinically silent for prolonged periods and become apparent only when abdominal viscera become displaced, incarcerated or functionally compromised. Nevertheless, because no previous diagnostic imaging or neonatal history was available, a remote acquired diaphragmatic tear could not be completely excluded.

In addition to the presumed chronic nature and anatomical location of the defect, this case was unusual because the herniated viscera consisted predominantly of hepatic tissue rather than gastrointestinal organs. In the largest available case series, diaphragmatic herniation was reported in both foals and adult horses, including adults ranging from 1 to 28 years of age; however, all horses were presented for colic of varying severity and duration [2]. Similarly, most reported equine diaphragmatic hernias involve incarceration of the small intestine or large colon, typically resulting in acute or recurrent colic and diagnosis during exploratory laparotomy [4, 6]. Other clinical contexts described in the literature include respiratory difficulty, exercise intolerance, lethargy, loss of condition or incidental post-mortem detection [4, 13, 14]. Congenital or developmental defects have also been reported mainly in foals, stillborn animals or young horses, sometimes in association with other congenital abnormalities or perinatal events [13, 14]. In contrast, the present case was dominated clinically by progressive neurological deterioration rather than colic or primary respiratory compromise. Hepatic herniation through a diaphragmatic defect has been described only rarely in horses [7], and the present case is notable for the predominance and extent of hepatic displacement associated with severe hepatobiliary dysfunction.

A central question in this case is how hepatic herniation resulted in generalised hepatic failure, given the considerable functional reserve of the equine liver. In horses, clinically significant hepatic dysfunction typically requires loss of more than 70–80% of functional hepatic mass [9]. In this context, long-distance transport may have acted as a precipitating stressor rather than as the primary cause of the diaphragmatic defect. Transport is associated with multiple physiological changes in horses, including activation of stress responses, increased cortisol concentrations, altered glucose and insulin dynamics, dehydration or haemoconcentration, changes in immune parameters, body weight loss and increased energy mobilisation [16–18]. These changes may be further amplified by reduced feed intake and prolonged confinement during transport. Although direct evidence linking transport to the onset of hepatic encephalopathy in horses with chronic liver disease is limited, transport is recognised as a physiological and metabolic stressor in this species. Therefore, it is plausible that transport-associated systemic stress could precipitate clinical decompensation in animals with critically reduced hepatic functional reserve, regardless of the underlying chronic hepatopathy. In the present case, this mechanism may have contributed to the transition from compensated chronic hepatic disease to overt hepatic encephalopathy [16–18].

The non-herniated right hepatic lobe also exhibited advanced histopathological changes, including bridging fibrosis and nodular regenerative remodelling, indicating that functional compromise was not limited to the incarcerated hepatic portion. Two interpretations must be considered. First, chronic constriction at the hernial ring may have progressively impaired hepatic perfusion and biliary drainage, generating haemodynamic disturbances that extended to the remaining parenchyma and contributed to secondary fibrotic remodelling over time. Chronic diaphragmatic herniation may result in persistent displacement and compression of abdominal viscera [19], while prolonged hepatic ischaemic injury may promote sustained fibrogenic responses and progressive parenchymal remodelling [20]. Second, a pre-existing hepatopathy of independent origin cannot be completely excluded. However, the topographic distribution of lesions favoured chronic herniation as the primary pathological event: severe atrophy, haemorrhagic necrosis, cholestasis, ductular proliferation, bile microliths and haemosiderin deposition were concentrated in the herniated lobes, whereas the non-herniated right lobe showed fibrotic and nodular remodelling without comparable necrosis or marked cholestasis. This anatomical pattern is more consistent with chronic vascular and biliary compromise related to incarceration than with a primary diffuse hepatopathy.

The absence of reported signs of chronic hepatic failure before the onset of hepatic encephalopathy may be explained, at least in part, by the large functional reserve of the equine liver. In horses, hepatic encephalopathy usually develops only when hepatic functional capacity is severely compromised [9]. Therefore, the non-herniated right hepatic lobe may have maintained sufficient residual function for a prolonged period, despite progressive remodelling, until a critical threshold of hepatic reserve was exceeded. The lack of reported weight loss or photosensitisation does not exclude chronic hepatic disease, as these signs are not invariably present and may depend on the type, severity, duration and functional consequences of the hepatopathy. For example, in chronic hepatotoxic plant poisoning affecting horses in Brazil, photosensitisation was not consistently observed despite severe hepatic disease [21]. In addition, the clinical history was obtained retrospectively, and subtle signs of chronic liver dysfunction may not have been recognised before acute neurological deterioration.

The clinicopathological findings supported clinically relevant hepatobiliary dysfunction. Markedly increased GGT activity, together with increased alkaline phosphatase ALP and AST activities and severe hyperbilirubinaemia, supported combined cholestatic and hepatocellular injury [11]. GGT is considered a sensitive indicator of cholestasis in horses; however, it should be interpreted in association with other biochemical and pathological findings rather than as an isolated marker of biliary obstruction [11, 20]. Hypertriglyceridaemia was interpreted cautiously, as it may have reflected negative energy balance or reduced feed intake during the acute clinical course, rather than hepatic metabolic failure alone [22]. However, impaired hepatic lipid metabolism may also have contributed, given the severe hepatobiliary dysfunction and lipid-type vacuolar degeneration observed histologically [23]. Hypercholesterolaemia was considered consistent with altered hepatobiliary and lipid metabolism, whereas hyperglycaemia was interpreted as most likely related to physiological stress, including the possible effect of recent transport-associated endocrine-metabolic changes [18]. Although serum bile acids, glutamate dehydrogenase and blood ammonia were not measured, the combination of severe hepatobiliary biochemical abnormalities, extensive hepatic lesions and Alzheimer type II astrocytes in the cerebral cortex supported the diagnosis of hepatic encephalopathy [9, 24]. Similar neuropathological changes have been reported in horses with chronic hepatopathies and hyperammonaemic encephalopathy [11, 25, 26]. Hyperbilirubinaemia may also have contributed to neurological dysfunction in the context of severe hepatic disease [27, 28]. In addition, the absence of inflammatory, necrotising, malacic or infectious lesions in the central nervous system made a primary neurological disease less likely [29].

The prognosis for horses with diaphragmatic hernia is guarded to poor, with survival rates of 23–46% reported in surgically managed cases [4, 6]. Cases involving chronic hepatic incarceration with advanced hepatobiliary dysfunction carry an even less favourable prognosis [8, 30]. Ante-mortem diagnosis in this case was challenging due to the absence of classical colic signs and the predominance of neurological abnormalities. Thoracic and abdominal ultrasonography would have been the most useful diagnostic tool, as it can identify pleural effusion, detect displacement of abdominal viscera into the thoracic cavity, and potentially visualise hepatic tissue within the thorax [6]. Peritoneal fluid analysis could also have provided additional information regarding hepatic or intestinal compromise. However, given the severity of the hepatic lesions documented at post-mortem examination, including advanced cirrhotic remodelling, cholestasis, and encephalopathy, the prognosis would likely have remained poor even with earlier recognition of the diaphragmatic defect.

The main limitations of this case are related to the incomplete ante-mortem clinical and diagnostic information available for review. Some clinical parameters were not fully recorded, including exact heart rate, respiratory rate and capillary refill time. In addition, selected laboratory tests that could have further supported the assessment of hepatic function and hepatic encephalopathy, such as serum bile acids, glutamate dehydrogenase, blood ammonia, urea and globulins, were not performed or were not available. Abdominal or thoracic ultrasonography was also not performed, which limited ante-mortem evaluation of hepatic position, pleural effusion and diaphragmatic integrity. Nevertheless, the combined clinical presentation, clinicopathological abnormalities, gross findings, hepatic histopathology and brain lesions compatible with hepatic encephalopathy provide relevant evidence that extensive chronic hepatic diaphragmatic herniation can contribute to severe hepatobiliary dysfunction and neurological decompensation in horses.

## Conclusion

This case documents an unusual presentation of equine diaphragmatic hernia in which extensive chronic hepatic incarceration, together with advanced hepatobiliary disease affecting both herniated and non-herniated hepatic tissue, was associated with severe hepatic failure and hepatic encephalopathy. The findings indicate that neurological abnormalities may predominate when hepatic functional reserve is critically compromised, even when classical abdominal signs are not reported. Diaphragmatic hernia with hepatic involvement should therefore be considered in the differential diagnosis of horses presenting with severe hepatopathy and neurological signs compatible with hepatic encephalopathy.

## Data Availability

The data supporting the findings of this study are included within the article. Additional information may be made available from the corresponding author upon reasonable request.
